# Effect of hydroxychloroquine on COVID-19 prevention in cancer patients undergoing treatment: a structured summary of a study protocol for a randomised controlled trial

**DOI:** 10.1186/s13063-020-04485-x

**Published:** 2020-06-26

**Authors:** Abolghasem Allahyari, Hossein Rahimi, Majid Khadem-Rezaiyan, Zahra Mozaheb, Mohsen Seddigh-Shamsi, Alireza Bary, Mostafa Kamandi, Sajad Ataei Azimi, Saeed Eslami HasanAbadi, Alireza Noferesti, Somayeh Sadat Shariatmaghani, Houshang Rafatpanah, Shohreh Khatami, Afshin Jabbar Imani, Hassan Mortazi, Mohammad Moeini Nodeh

**Affiliations:** 1 Division of Hematology and Oncology, Department of Internal Medicine, Faculty of Medicine, Mashhad University of Medical Sciences, Mashhad, Iran; 2 Community Medicine, Clinical Research Development Unit, Faculty of Medicine, Mashhad University of Medical Sciences, Mashhad, Iran; 3 Department of Internal Medicine, Faculty of Medicine, Mashhad University of Medical Sciences, Mashhad, Iran; 4 Medical Informatics, Pharmacy School, Mashhad University of Medical Sciences, Mashhad, Iran; 5 Immunology, Immunology Department, Faculty of Medicine, Mashhad University of Medical Sciences, Mashhad, Iran; 6 Internal Medicine, Department of Internal Medicine, Faculty of Medicine, Mashhad University of Medical Sciences, Mashhad, Iran; 7 Hematology and Oncology Section, Internal Medicine Department, Ghaem Hospital, Ahmadabad Ave, Shariati Sq, Mashhad, Iran

**Keywords:** COVID-19, Randomised controlled trial, Protocol, Hydroxychloroquine, Acute Lymphoid Leukemia, Acute Myeloid Leukemia, Breast cancer, Colon cancer, Prophylaxis

## Abstract

**Objectives:**

In this study, we investigate the effect of hydroxychloroquine on the prevention of Novel Coronavirus Disease (COVID-19) in cancer patients being treated.

**Trial design:**

This is a multi-centre, two-arm, parallel-group, triple-blind, phase 2-3 randomised controlled trial.

**Participants:**

All patients over the age of 15 from 5 types of cancer are included in the study. Patients with acute lymphoid and myeloid leukemias in the first line treated with curative intent, patients with high-grade non-Hodgkin's lymphoma treated with leukemia protocols and patients with non-metastatic breast and colon cancer in the first line of treatment will enter the study. The exclusion criteria will include known sensitivity to Hydroxychloroquine, weight below 35 kilograms, history of retinopathy, history of any cardiac disease, acute respiratory tract infection in the last 2 months, having COVID-19 in the first two weeks of entering the trial, having Diabetes Mellitus, having an immuno-suppressive disease other than cancer, having chronic pulmonary disease and taking immuno-suppressant drug other than chemotherapeutic agents for current cancer. This study is performed in five academic centres affiliated to Mashhad University of Medical Sciences, Mashhad, Iran.

**Intervention and comparator:**

Patients are randomly assigned to two groups; one being given hydroxychloroquine and the other is given placebo. During two months of treatment, the two groups are treated with either hydroxychloroquine (Amin® Pharmaceutical Company, Isfahan, Iran) or placebo (identical in terms of shape, colour, smell) as a single 200 mg tablet every other day. Patients will be monitored for COVID-19 symptoms during the follow-up period. If signs or symptoms occur (fever, cough, shortness of breath), they will be examined and investigated with a high-resolution computed tomography (CT) scan of the lungs, COVID-19 specific IgM, IgG antibody assay and a nucleic acid amplification test (NAT) for the SARS-CoV-2 virus.

**Main outcomes:**

The primary end point of this study is to investigate the incidence of COVID-19 in patients being treated for their cancer over a 2-month period.

**Randomisation:**

Randomisation will be performed using randomly permuted blocks. By using an online website (www.randomization.com) the randomization sequence will be produced by quadruple blocks. The allocation ratio in intervention and control groups is 1:1.

**Blinding (masking):**

Participants and caregivers do not know whether the patient is in the intervention or the control group. The outcome assessor and the data analyst are also blinded to group assignment.

**Numbers to be randomised (sample size):**

The calculated total sample size is 60 patients, with 30 patients in each group.

**Trial Status:**

The trial began on April 14, 2020 and recruitment is ongoing. Recruitment is anticipated to be completed by June 14, 2020 There has been no change in study protocol since approval, protocol version 1 was approved April 12, 2020.

**Trial registration:**

This trial has been registered by the title of “*Effect of Hydroxychloroquine on Novel Coronavirus Disease (COVID-19) prevention in cancer patients under treatment*” in Iranian Registry of Clinical Trials (IRCT) with code “IRCT20200405046958N1”, https://www.irct.ir/trial/46946. Registration date is April 14, 2020.

**Full protocol:**

The full protocol is attached as an additional file, accessible from the Trials website (Additional file 1). In the interest in expediting dissemination of this material, the familiar formatting has been eliminated; this Letter serves as a summary of the key elements of the full protocol.

## Supplementary information

**Additional file 1.**

## Data Availability

The final dataset of the trial will be available upon request from the primary investigator via e-mail at moeininm@mums.ac.ir, after obtaining the permission of the Regional Ethics Committee.

