# Supplementary material for: Effect of hydroxychloroquine on COVID-19 prevention in cancer patients undergoing treatment: a structured summary of a study protocol for a randomised controlled trial
Source: Trials. 2020 Jun 26;21:575. doi: 10.1186/s13063-020-04485-x (PMC7316578; doi:10.1186/s13063-020-04485-x)
Supplement: Supplementary file 1 — Additional file 1. [file 13063_2020_4485_MOESM1_ESM.pdf]

بررسی تاثیر مصرف هیدروکسی کلرکین بر پیشگیری از ابتلا به COVID-19 در بیماران مبتلا به سرطان در حال درمان

مقدمه

اهمیت موضوع

بروز بیماری جدیدی با نام COVID-19 ناشی از یک ویروس از خانواده کرونا ویروس به نام SARS-nCoV-2 در تمام دنیا باعث مشکلات شدید بهداشتی و اقتصادی شده است. این بیماری اواخر سال 2019 میلادی برای اولین بار در شهر ووهان Wuhan مرکز استان هوبه‌ی Hubei چین به صورت پنومونی گزارش شد. طی ماههای اخیر و سرعت گسترش بالای این بیماری که باعث ایجاد پاندمی ظرف مدت کمتر از 3 ماه از پیدایش آن شد، نگرانی های بسیاری در سیستم های بهداشتی درمانی کشورهای مختلف ایجاد کرده است. جدید بودن و ناشناخته بودن ویروس از یک طرف و سرعت بالای گسترش آن همه را غافلگیر کرده است.

تمام شواهد فعلی حاکی از استعداد بالاتر بیماران دارای نقص ایمنی به ابتلای به این بیماری هستند. از موارد بسیار شایع ایجاد کننده نقص ایمنی می توان به سرطان و درمان های آن اشاره کرد. امروزه سرطان به عنوان عامل بخش بزرگی از مرگ و میر و ابتلا بیماران در دنیا چالش بهداشتی مهمی محسوب می شود. بیمار مبتلا به سرطان به دو دلیل دچار ضعف سیستم ایمنی است. اولین عامل خود سرطان است که به خصوص در برخی از بدخیمی های هماتولوژیک یعنی لوسمی های حاد به شدت سیستم ایمنی بیمار را تضعیف می کند. نقش این عامل در انواع بدخیمی های ارگانهای تو پر (solid tumors) کم رنگ تر است. اما عامل دومی که تاثیر بسیار بیشتری روی سیستم ایمنی دارد درمان سرطان است. استفاده از انواع داروهای سایتوتوکسیک (Cytotoxic) در رژیم های شیمی درمانی سرطان و داروهای ایمونوتراپی (Immunotherapy) با دست کاری سیستم ایمنی باعث تضعیف بیشتر ایمنی و قدرت مقابله بیمار با عفونت ها می شود. در این شرایط گاهی سیستم ایمنی به قدری ضعیف می شود که حتی فلور نرمال بدن خود بیمار برایش تهدیدی بزرگ محسوب می شود.

بروز بیماری ویروسی COVID-19 برای بیماران سرطانی و پزشکان دخیل در امر درمان سرطان باعث نگرانی های زیادی شده است. ترس از ابتلا به عفونت ویروسی ای که تا کنون هیچ درمان موثر و اختصاصی نداشته و می تواند به سرعت یک فرد دچار ضعف ایمنی را از پای درآورد باعث اختلال های گسترده ای در درمان بیماران سرطانی شده است. مراکز بزرگ و معتبر تولید کننده دستورالعمل ها و توصیه های درمانی سرطان در مقطع کنونی پیشنهادات مختلفی ارائه نموده اند. نتیجه بیشتر این پیشنهادات نیز اغلب چیزی جزء تاخیر درمان در بسیاری از بیماران نیازمند درمان یا استفاده از درمان های کمتر موثر نبوده است. نتایج این اقدامات در بلند مدت مشخص خواهند شد.

اتخاذ تدابیر درمانی جدید به نحوی که بتوان کامل ترین درمان اختصاصی سرطان را در زمان مناسب به بیمار ارائه نمود بسیار اهمیت دارد. آن طور که پیش بینی شده است این ویروس جدید و عوارض آن به زودی به پایان کار نخواهند رسید و حتی ممکن است مهمان همیشگی بشر باشند. در حال حاضر بیشتر امکانات و توان نیروهای درمانی در مراکز بهداشتی و درمانی به سمت کنترل بیماری COVID-19 معطوف شده و متاسفانه بسیاری از دیگر شاخص های مهم بهداشت و سلامت تحت تاثیر قرار گرفته است.

راه کار

رعایت کردن تمام پرتکل های پیشگیری کنونی از جمله ایزوله کردن، ضد عفونی کردن و شستشوی مکرر دستها و سطوح، استفاده از ماسک های مختلف فیلتر دار و یا ساده به سبب مقتضیات درمانی بیماران سرطانی نتایج مطلوبی در پیشگیری از

ابتلای ایشان به COVID-19 نداشته است. لذا بر آن شدیم تا علاوه بر رعایت نکات معلوم شده فعلی در پیشگیری از انتشار و ابتلای به ویروس SARS-nCoV-2 در بیماران سرطانی روش جدیدی برای کمتر کردن احتمال ابتلای ایشان را مورد مطالعه قرار دهیم.

مطالعات مختلفی در گذشته اثرات ضد انگلی و ضد ویروسی داروی هیدروکسی کلرکین را نشان داده اند (1-4). این دارو به سهولت در دسترس بوده و مصرف آن به شکل قرص خوراکی بسیار راحت و عوارض قابل پایش و اندکی دارد. تداخلات دارویی شناخته شده ای هم با داروهای شیمی درمانی مختلف، ایمنی درمانی و یا هدف درمانی ندارد. نشان داده شده که هیدروکسی کلرکین اثرات مهاری روی عفونتهای ویروسی مختلفی از جمله آدنوویروس، ویروس آنفلوآنزا و حتی HIV1 دارد. این اثر از طریق پیشگیری از ورود ویروس به سلول اعمال می شود. هیدروکسی کلرکین اثرات خوبی هم روی سایر ارگانیسم های داخل سلولی مانند مالاریا و حتی باکتری داخل سلولی کوکسیلا بورنتی داشته است. در مطالعات دیگری ابتدا در محیط آزمایشگاه و سپس در بدن انسان اثرات مثبت ضد ویروسی این دارو روی ویروسهای خانواده کرونا از جمله کرونا ویروس جدید نشان داده شده است (5-11). اخیرا نیز سازمان غذا و داروی ایالات متحده آمریکا (FDA) مجوز اضطراری استفاده از هیدروکسی کلرکین برای برخی از بیماران بستری COVID-19 در بیمارستان و یا استفاده در غالب مطالعات کارآزمایی بالینی را صادر کرده است.

دارو، مکانیسم اثر، ایمنی و عوارض

داروی هیدروکسی کلرکین سولفات مشتق کلرکین فسفات بوده که کاربرد اصلی و تاریخی آن درمان مالاریا است. مکانیسم اثر دارو در مالاریا ایجاد تداخل با عملکرد واکوئول هضمی انگل بوسیله افزایش PH و تداخل در تخریب هموگلوبین، مهار جذب نوتروفیل ها و کموتاکسی ائوزینوفیل ها و تضعیف واکنش های آنتی ژن-آنتی بادی با واسطه کمپلمان است.

اما مطالعات مختلفی اثرات ضد ویروسی دارو را نشان داده اند. مکانیسم اثر ضد ویروسی کاملا شناخته نشده است اما تغییر PH سطح غشای سلولی که مانع اتصال ویروس به سلول می شود به عنوان یک مکانیسم مطرح شده است. هیدروکسی کلرکین همچنین می تواند تکثیر محتوای نوکلئیک اسیدی، گلیکوزیلاسیون پروتئین ها و ساخت و آزاد سازی ویروس را مهار کند (12).

هیدروکسی کلرکین پروفایل عوارض و ایمنی قابل قبولی دارد و مهم ترین عوارض شامل طولانی شدن فاصله QT در قلب و ایجاد آریتمی ثانویه به آن و رتینوپاتی در چشم است. سایر عوارض کمتر شایع (به صورت گزارش موردی) عبارتند از عوارض هماتولوژیک شامل آگرانولوسیتوز، آنمی، آنمی آپلاستیک و نارسای مغز استخوان، همولیز (در بیماران دچار کمبود G6PD) و لکوپنی و ترومبوسیتوپنی، عوارض کبدی شامل بالا رفتن ترانس آمیناز های کبدی، عوارض گوارشی شامل بی اشتهایی، درد شکم، اسهال و تهوع و استفراغ، عوارض متابولیک و اندوکرین شامل هیپوگلیسمی، تشدید پورفیری و کاهش وزن، عوارض پوستی شامل راش، تغییر رنگ مو، آلوپسی، خارش و کهیر و عوارض عصبی شامل گیجی، آتاکسی، خستگی، سردرد و تحریک پذیری است.

در مدت سپری شده از بحران جهانی COVID-19 در بسیاری از پرتکل های درمانی بیماران بستری و حتی سرپایی از جمله در ایران، هیدروکسی کلرکین برای مقطعی استفاده شد. هنوز گزارش دقیقی از اثرات مفید یا مضر آن ارائه نشده است. اما آنچه مشهود بود تحمل خوب دارو حتی در بیماران بسیار بد حال و در حال مصرف داروهای متعدد است.

تداخلات دارویی هیدروکسی کلرکین با تمام داروهایی که در درمان سرطان بیماران مورد مطالعه قرار است مورد استفاده قرار گیرد، مورد بررسی قرار گرفت و هیچ تداخل اثر شناخته شده ای وجود ندارد.

طراحی مطالعه

اهداف

این مطالعه یک کارآزمایی بالینی تصادفی شده چند مرکزی با هدف زیر است.

هدف اصلی:

- تعیین اثر مصرف هیدروکسی کلرکین بر پیشگیری از ابتلا به COVID-19 در بیماران مبتلا به سرطان در حال درمان

گروه های مورد مطالعه

این مطالعه بر روی بیماران مبتلا به سرطانی که در شرایط بحران بیماری COVID-19 کماکان مجبور به ادامه درمان بیماری خود بوده انجام می شود.

سرطان های مختلف از نظر میزان مخاطره ایجاد کننده برای ابتلا به عفونت به سه گروه پر خطر، خطر متوسط و کم خطر تقسیم بندی می شوند. به خاطر ملاحظات آماری تصمیم بر این شد تا از دو گروه کم و پر خطر بیماران انتخاب و وارد مطالعه شوند. در گروه کم خطر دو سرطان بسیار شایع پستان و کولون انتخاب شدند. ماهیت بیماری های گروه کم خطر و رژیم های درمانی آن ها از نظر ایجاد نقص ایمنی تفاوت بارزی با هم نداشته اما با هدف امکان تعمیم نتایج دو سرطان شایع این گروه انتخاب شدند. در گروه پر خطر لوسمی های حاد لنفوئیدی و میلوئیدی و لنفوم های غیر هوچکینی با درجه بدخیمی بالا که با پرتکل های لوسمی درمان می شوند، برای ورود به مطالعه انتخاب شدند.

معیارهای ورود:

1. بیماران مبتلا به سرطان خونی حاد لنفوئیدی قابل علاج
2. بیماران مبتلا به سرطان خونی حاد میلوئیدی قابل علاج
3. بیماران مبتلا به سرطان غدد لنفاوی غیر هوچکینی با درجه بدخیمی بالا قابل علاج که با رژیم مشابه لوسمی درمان می شوند.
4. بیماران مبتلا به سرطان پستان قابل علاج
5. بیماران مبتلا به سرطان روده بزرگ قابل علاج
6. سن بیشتر از 15 سال

معیارهای خروج

1. حساسیت به داروی هیدروکسی کلرکین
2. وزن زیر 35 کیلو گرم
3. سابقه رتینوپاتی
4. سابقه هر نوع بیماری قلبی
5. عفونت تنفسی حاد طی 2 ماه اخیر
6. ابتلا به بیماری کرونا ویروس جدید در دو هفته ابتدای ورود به طرح
7. دیابت شیرین
8. بیماری ایجاد کننده نقص ایمنی غیر از سرطان
9. بیماری مزمن ریوی
10. مصرف داروی سرکوب گر ایمنی غیر از شیمی درمانی سرطان

تصادفی سازی

بعد از انتخاب بیماران بر اساس معیار های ورود و خروج و اخذ رضایت نامه آگاهانه، اختصاص تصادفی ایشان به یکی از دو بازوی مداخله یا کنترل انجام می شود.

نحوه تصادفی سازی با استفاده از بلوک های تصادفی خواهد بود. با استفاده از وب سایت www.randomization.com توالی تصادفی با استفاده از بلوک های 4 تایی تولید خواهد شد.

کور سازی

این مطالعه به صورت سه سو کور انجام می شود. بیمار و پزشک معالج و تحلیل گر داده ها از گروه دارو یا دارو نما اطلاعی ندارند.

روش اجرا

بیماران در این مطالعه در دو بازوی مداخله یا کنترل قرار گرفته و به طور تصادفی و کور شده به ترتیب دارو و دارو نما دریافت خواهند کرد.

دوز انتخاب شده داروی هیدروکسی کلرکین یک عدد قرص 200 میلی گرمی به صورت روز در میان است. علت انتخاب این دوز در نظر گرفتن نیمه عمر طولانی، متابولیسم نا معلوم، تحمل گوارشی کمتر در بیماران تحت درمان سرطان و به حداقل رساندن عوارض احتمالی در عین حفظ تاثیر دارو بوده است.

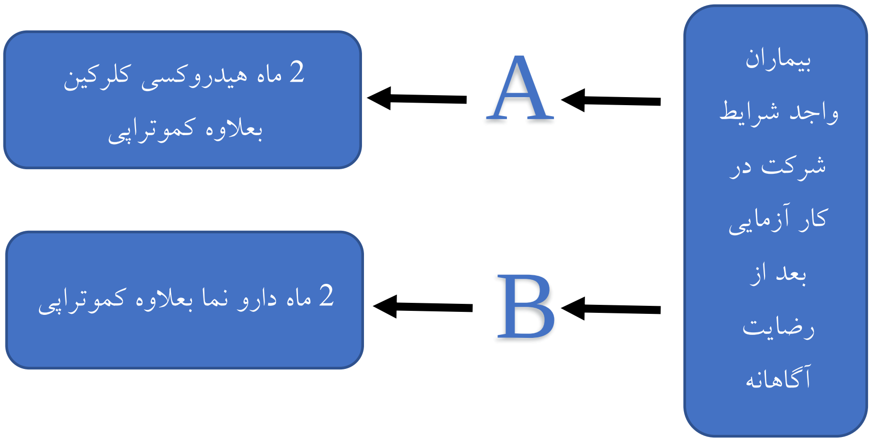

در گروه مداخله بیماران در کنار درمان سرطان خود برای مدت دو ماه داروی هیدروکسی کلرکین را به صورت روز در میان یک عدد قرص 200 میلی گرمی مصرف می کنند. در گروه کنترل نیز علاوه بر درمان سرطان دارو نما به بیمار داده می شود. درمان سرطان بیمار بر اساس پرتکل های استاندارد و به انتخاب پزشک معالج و بیمار و بدون دخالت محقق صورت می گیرد.

بیماران در طول مطالعه از نظر علائم ابتلا به بیماری COVID-19 پایش می شوند. علائم بیماری به ایشان آموزش داده می شود و با بروز هر گونه علائم مشکوک، بیمار به کلینیکی که برای این کار اختصاص داده شده ارجاع می شود. در این کلینیک برای بیمار بعد از شرح حال و معاینه توسط متخصص داخلی در صورت ظن بالینی به COVID-19 بررسی های پاراکلینیکی تکمیلی شامل بررسی درصد اشباع اکسیژن خون شریانی، CRP کمی، شمارش کامل خون (CBC) به همراه سی تی اسکن ریه با قدرت تفکیک بالا (Lung High Resolution CT Scan)، آزمایش سرولوژی آنتی بادی اختصاصی COVID-19 از هر دو نوع IgM و IgG و بررسی نوکلئیک اسید ویروس SARS CoV-2 به روش RT-PCR (Reverse Transcriptase Polymerase Chain Reaction) از ترشحات نازوفارنکس انجام می شود.

بیمار گیری

از بین بیماران مراجعه کننده به کلینیک های هماتولوژی انکولوژی سرپایی و بخش های بستری هماتولوژی انکولوژی دو بیمارستان اصلی دانشگاهی در دانشگاه علوم پزشکی مشهد، یعنی بیمارستان های امام رضا (ع) و قائم (عج) بیماران مبتلا به سرطان های پستان، کولون، لوسمی حاد لنفوئیدی، لوسمی حاد میلوئیدی و لنفوم های غیر هوچکینی با درجه بدخیمی بالا که بنابر نظر پزشک معالج باید درمان مشابه لوسمی دریافت کنند به محققین معرفی می شوند. تطبیق بیماران ارجاعی با معیارهای ورود و خروج توسط محقق انجام و مراحل اجرای پژوهش برای بیمار به زبان ساده توضیح داده می شود. در صورت موافقت بیمار فرم رضایت نامه آگاهانه در دو نسخه به تایید بیمار رسانده می شود. یک نسخه نزد خود بیمار و نسخه دوم نزد پژوهشگر نگهداری می شود.

پاکت های تصادفی سازی بیماران که توسط اپیدمیولوژیست آماده شده اند به همراه بسته هایی حاوی 30 عدد قرص بدون اینکه مشخص باشد کدام دارو یا دارو نما است در اختیار پزشک معالج قرار می گیرد. پاکت های تصادفی سازی به گونه ای انتخاب شده اند که محتوای داخل آن مشخص نباشد. داخل هر پاکت یک برگه تعیین کننده بازوی درمانی بیمار وجود دارد. پزشک معالج که در خصوص اینکه کدام بازو مداخله و کدام کنترل است کور شده، به طور اتفاقی برای هر بیمار یک پاکت انتخاب و بسته قرص مربوطه را نیز به بیمار تحویل می دهد.

پایش عوارض

در طول مدت مطالعه بیماران از نظر عوارض قلبی با انجام نوار قلب در اولین جلسه و سپس تکرار آن در هر جلسه شیمی درمانی و یا حداکثر هر 4 هفته پایش می شوند. مهم ترین نکته ای که در نوار قلب مد نظر است محاسبه فاصله QTc است که اگر در اولین جلسه این فاصله بیشتر از 500 میلی ثانیه باشد بیمار وارد طرح نخواهد شد. اگر در بررسی ها میانه درمان فاصله QTc طولانی تر از 500 میلی ثانیه شده باشد دارو/دارو نما قطع و بیمار از مطالعه خارج می شود و به متخصص قلب ارجاع می شود .

در بدو ورود به مطالعه از بیماران شرح حال جامع مشکلات چشمی گرفته می شود و در صورت فقدان هر مشکل چشمی بیمار در هر ویزیت از نظر بروز علائم جدید با شرح حال ارزیابی می شود. در صورت بروز علامت جدید معاینه توسط چشم پزشک درخواست خواهد شود. با توجه به اینکه عوارض چشمی هیدروکسی کلرکین در مصرف دراز مدت و اغلب بعد از یک سال گزارش شده است معاینه دوره ای در بیماران بدون علامت در نظر گرفته نشد.

درباره سایر عوارض کمتر شایع دارو مانند عوارض هماتولوژیک، پوستی و گوارشی بیمار توسط پزشک معالج تحت پایش است. علاوه بر آن یک نفر پزشک متخصص داخلی از طرف تیم تحقیق صرفا برای پایش عوارض مختلف دارو به بیماران معرفی و شماره تلفن وی در اختیار بیماران قرار داده شده تا هر زمان که لازم بود تلفنی یا حضوری مورد بررسی قرار گیرند.

نحوه بررسی هدف مطالعه (ابتلا به عفونت COVID-۱۹)

در طول مطالعه بیماران از نظر بروز علائم عفونت تنفسی پایش می شوند و در صورت بروز علائم تب، تنگی نفس، سرفه، بدن درد و میالژی تحت بررسی ابتلا به عفونت COVID-19 قرار می گیرند. در اولین قدم شرح حال جامع از بیمار اخذ می شود. معاینه فیزیکی هدفمند برای علل عفونی با تمرکز بر COVID-19 توسط پزشک متخصص داخلی یا پزشک معالج مدیکال انکولوژیست انجام می شود. بررسی درصد اشباع اکسیژن خون شریانی جزئی از معاینه فیزیکی بیمار است. تست های آزمایشگاهی غربالگری عفونت COVID-19 شامل CBC، CRP و سی تی اسکن با قدرت تفکیک بالا از ریه ها برای بیمار مشکوک به عفونت درخواست می شود. در صورت غیر طبیعی بودن هر یک از موارد ذکر شده در قدم بعدی آنتی بادی اختصاص از هر دو نوع IgG و IgM با روش الایزا و بررسی نوکلئیک اسید ویروس با RT-PCR درخواست می شود.

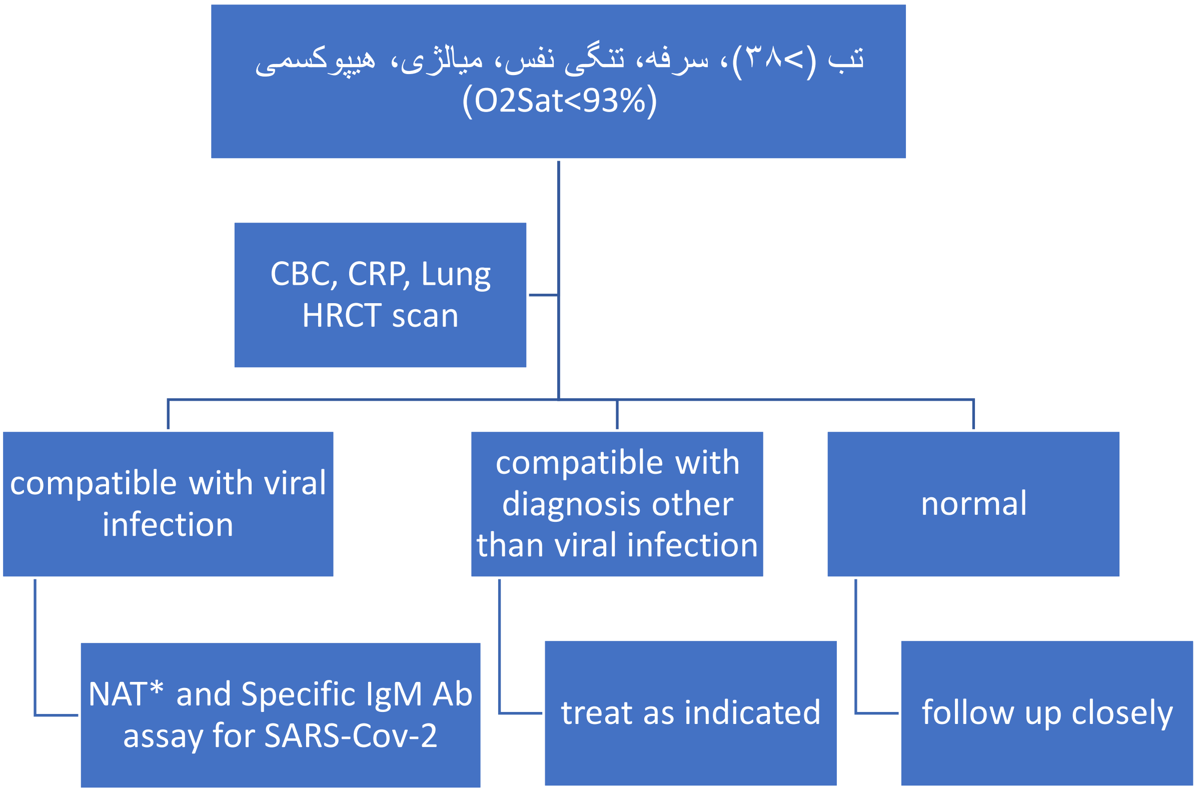

❖ Nasopharyngeal/oropharyngeal swab for nucleic acid amplification test by RT-PCR.

اگر علاوه بر علائم بالینی مطرح کننده بیماری COVID-19 درگیری ریوی در سی تی اسکن به طور تیپیک (با تایید رادیولوژیست و پولمونولوژیست) مطرح کننده عفونت ویروسی باشد و تستهای سرولوژی و NAT منفی باشند، بیمار مورد مشکوک قلمداد شده و تحت درمان های مناسب قرار می گیرد.
اگر آنتی بادی اختصاصی ویروس نوع IgM یا تست NAT مثبت باشد بیمار مورد قطعی COVID-19 تلقی شده و تحت درمان های مناسب قرار خواهد گرفت.
اگر تمام آزمونهای پاراکلینکی فوق منفی بوده و فقط تست آنتی بادی اختصاصی نوع IgG مثبت شود بیمار به عنوان مبتلا شده قبلی تلقی شده و از مطالعه خارج می شود.

روش تحلیل داده ها

تحلیل داده ها با استفاده از نرم افزار SPSS نسخه 16 انجام خواهد شد. توصیف متغیرهای کمی توسط میانگین و انحراف معیار و توصیف متغیرهای کیفی توسط فراوانی و درصد فراوانی خواهد بود. مقایسه متغیرهای کمی در دو گروه مداخله و کنترل توسط آزمون تی دانشجویی و در صورت عدم تبعیت از توزیع نرمال از آزمون من ویتنی استفاده خواهد شد. مقایسه متغیرهای کیفی در دو گروه مذکور توسط آزمون مجذور کای و در صورت لزوم تست دقیق فیشر خواهد بود. مقایسه های درون گروهی توسط آزمون تی زوجی و در صورت عدم تبعیت از توزیع نرمال توسط آزمون ویلکاکسون خواهد بود. تمامی آزمون ها دو طرفه و سطح معنی داری کمتر از 0/05 خواهد بود.

منابع

1. SPERBER K, KALB TH, STECHER VJ, BANERJEE R, MAYER L. Inhibition of human immunodeficiency virus type 1 replication by hydroxychloroquine in T cells and monocytes. AIDS research and human retroviruses. 1993 Jan;9(1):91-8.
2. Wang LF, Lin YS, Huang NC, Yu CY, Tsai WL, Chen JJ, Kubota T, Matsuoka M, Chen SR, Yang CS, Lu RW. Hydroxychloroquine-inhibited dengue virus is associated with host defense machinery. Journal of Interferon & Cytokine Research. 2015 Mar 1;35(3):143-56.
3. Colson P, Rolain JM, Lagier JC, Brouqui P, Raoult D. Chloroquine and hydroxychloroquine as available weapons to fight COVID-19. Int J Antimicrob Agents. 2020 Mar 4;105932.
4. Al-Bari MA. Chloroquine analogues in drug discovery: new directions of uses, mechanisms of actions and toxic manifestations from malaria to multifarious diseases. Journal of Antimicrobial Chemotherapy. 2015 Jun 1;70(6):1608-21.
5. Wang M, Cao R, Zhang L, Yang X, Liu J, Xu M, Shi Z, Hu Z, Zhong W, Xiao G. Remdesivir and chloroquine effectively inhibit the recently emerged novel coronavirus (2019-nCoV) in vitro. Cell research. 2020 Mar;30(3):269-71.
6. Gao J, Tian Z, Yang X. Breakthrough: Chloroquine phosphate has shown apparent efficacy in treatment of COVID-19 associated pneumonia in clinical studies. BioScience Trends. 2020.
7. Chinese Clinical Trial Registry. http://www.chictr.org.cn/searchproj.aspx?title=%E6%B0%AF%E5%96%B9&officialname=&subjectid=&secondaryid=&applier=&studyleader=&ethicalcommitteesanction=&sponsor=&studyailment=&studyailmentcode=&studytype=0&studystage=0&studydesign=0&minstudyexecutetime=&maxstudyexecutetime=&recruitmentstatus=0&gender=0&agreetosign=&secsponsor=&regno=&regstatus=0&country=&province=&city=&institution=&institutionlevel=&measure=&intercode=&sourceofspends=&createyear=0&isuploadrf=&whetherpublic=&btngo=btn&verifycode=&page=1.
8. Pourdowlat G, Panahi P, Pooransari P, Ghorbani F. Prophylactic Recommendation for Healthcare Workers in COVID-19 Pandemic. Advanced Journal of Emergency Medicine. 2020 Mar 27.
9. Post-exposure Prophylaxis / Preemptive Therapy for SARS-Coronavirus-2 (COVID-19 PEP). ClinicalTrials.gov Identifier: NCT04308668

10. Hydroxychloroquine Chemoprophylaxis in Healthcare Personnel in Contact With COVID-19 Patients (PHYDRA Trial) (PHYDRA). ClinicalTrials.gov Identifier: NCT04318015.
11. Treatment of COVID-19 Cases and Chemoprophylaxis of Contacts as Prevention (HCQ4COV19). ClinicalTrials.gov Identifier: NCT04304053.
12. Yao X, Ye F, Zhang M, Cui C, Huang B, Niu P, Liu X, Zhao L, Dong E, Song C, Zhan S. In vitro antiviral activity and projection of optimized dosing design of hydroxychloroquine for the treatment of severe acute respiratory syndrome coronavirus 2 (SARS-CoV-2). Clinical Infectious Diseases. 2020 Mar 9.
